# Icatibant as an early rescue therapy in hypovolemic shock with converting enzyme inhibitor treatment

**DOI:** 10.1186/s13054-017-1857-0

**Published:** 2017-11-02

**Authors:** Hélène Charbonneau, Marie Buléon, Benoit Richard, Nicolas Mayeur

**Affiliations:** 1 Groupe d’Anesthésie-Réanimation Chirurgie cardio-thoracique et vasculaire, Clinique Pasteur, 45 av Lombez, BP 27617 31076 Toulouse Cedex 3, France; 2 Institut National de la Santé et de la Recherche Medicale (INSERM), U1048, Institut des maladies métaboliques et cardiovasculaires I2MC, 1 Avenue Jean Poulhès, BP 84225 31432 Toulouse Cedex 4, France; 3 Université Toulouse III Paul Sabatier, Toulouse, France; 4 Pôle Anesthésie-Réanimation; CHU Rangueil, Avenue Jean Poulhès, F-31059 Toulouse Cedex 9, France

Activation of the renin-angiotensin system is a key adaptive response to hypovolemia aiming at maintaining mean arterial pressure, cardiac output, and organ perfusion. Angiotensin converting enzyme inhibitor (ACEI) therapy, widely prescribed to treat chronic hypertension, chronic heart disease, or diabetic nephropathy, blunts this adaptive mechanism [1]. These treatments may therefore put patients at risk of life-threatening complications after general anesthesia or hypovolemia [2, 3]. Because the pharmacological effects of ACEI therapy are partly mediated by bradykinin B2 receptor (B2R) activation, we hypothesized that acute B2R blockade would be beneficial in this setting. We previously showed that acute B2R blockade by icatibant, a specific B2R antagonist, significantly improves hemodynamics in a murine model of mild pressure and volume-targeted hemorrhagic shock under ACEI treatment (with 0.3 ml of blood withdrawn) [4]. In the present study, we tested the hypothesis that acute B2R blockade increases survival of ACEI-treated mice in severe hemorrhagic shock. To confirm this hypothesis, we induced severe volume-targeted hemorrhagic shock by 40% blood spoliation (i.e., 0.6 ml).

First, we confirmed our previous results regarding the beneficial effect of icatibant to prevent the deleterious hemodynamic consequences of ACEIs in shocked mice. In fact, the mean arterial blood pressures during shock in the ACEI group were significantly lower compared to the control and ACEI group treated with icatibant (25 [25–75% interquartile range (IQR), 21–29] mmHg vs 38 [25–75% IQR, 33–43] mmHg and 36 [25–75% IQR, 35–38] mmHg, respectively; *p* < 0.001). Second, we showed that survival in the control and ACEI groups treated with icatibant was significantly higher (81.8 and 90.9%, respectively) than in the ACEI group (36.4%) (*p* < 0.01). Of note, poor outcome was mostly observed in ACEI-treated mice while control untreated mice had good outcomes. Figure 1 describes the protocol design (Fig. 1a) and shows the survival curve from the beginning of shock to sacrifice (Fig. 1b). The life-saving benefit of B2R blockade was quickly achieved and sustained with a single subcutaneous injection of icatibant. This injection was given only prior to shock. None of the group received conventional vasopressors. However, efficacy of vasopressors has been challenged in this setting [1].

**Fig. 1:**
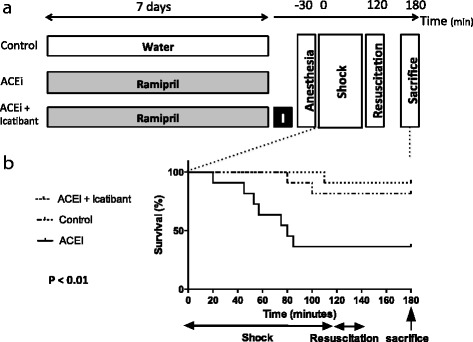
**a** Protocol design. **b** Survival curve in volume-targeted hemorrhagic shock. Hemorrhagic shock was induced with the same protocol as previously described [4]. In this study, the main difference was the withdrawn volume of blood (0.6 ml vs 0.3 ml) to induce volume-targeted hemorrhagic shock. Briefly, the animals were anesthetized with ketamine and xylazine and intubated using an intratracheal cannula. The left jugular vein and femoral artery were catheterized, and ketamine was continuously infused until the end of the shock procedure. A volume of 0.6 ml of blood was withdrawn through the femoral arterial line in order to induce hemorrhagic shock. The blood was re-infused at the end of the 2-h period of shock with the addition of an equal volume of lactated Ringer’s solution to ensure appropriate fluid volume restoration. The mice were sacrificed 1 h after the shock procedure. An ACEI (ramipril) was given at a dose of 1 mg/kg per day in drinking water (at a concentration of 5 μg/ml) during the 7 days prior to shock. Icatibant was subcutaneously administered at a dose of 250 μg/kg at the same time as anesthesia. *Control*, control shocked mice; *ACEI*, shocked mice treated with angiotensin-converting enzyme inhibitor; *ACEI + icatibant*, shocked mice treated with both ACEIs and a single injection of icatibant at the same time as the administration of anesthesia. *N* = 11 per group. Qualitative variables were described using the median (25–75% interquartile range). Comparisons between groups were performed using the nonparametric Kruskal–Wallis one-way analysis of variance followed by the post hoc Dunn’s test. Survival curves were computed according to Kaplan–Meier and compared with the log-rank test. Analyses were performed using GraphPad Prism 4 (GraphPad Software Inc., San Diego, CA, USA). Survival in both ACEI + icatibant and control groups was significantly higher than in the ACEI group (*p* < 0.01)

The results of this proof-of-concept study suggest that very early administration of icatibant may limit the consequences of hemorrhagic shock in patients treated with ACEIs before resuscitation could be initiated. Further research is needed with respect to dose and timing of icatibant administration and comparison with conventional vasopressors.

## Abbreviations

ACEI: Angiotensin converting enzyme inhibitor
B2R: Bradykinin B2 receptor

## References

[1] Mets B (2013). Management of hypotension associated with angiotensin-axis blockade and general anesthesia administration. J Cardiothorac Vasc Anesth.

[2] Mets B (2015). To stop or not?. Anesth Analg.

[3] Auron M, Harte B, Kumar A, Michota F (2011). Renin-angiotensin system antagonists in the perioperative setting: clinical consequences and recommendations for practice. Postgrad Med J.

[4] Charbonneau H, Buléon M, Minville V, Faguer S, Girolami J-P, Bascands J-L (2016). Acute bradykinin receptor blockade during hemorrhagic shock in mice prevents the worsening hypotensive effect of angiotensin-converting enzyme inhibitor. Crit Care Med.

